# Imaging the PM/AICD patient; fancy or fanatical?

**DOI:** 10.1186/1532-429X-15-S1-T3

**Published:** 2013-01-30

**Authors:** June Yamrozik, Mark Doyle, Ronald B Williams, Sahadev T Reddy, Moneal Shah, Geetha Rayarao, Diane A Vido, Robert W Biederman

**Affiliations:** 1 Cardiac MRI, Allegheny General Hospital, Pittsburgh, PA, USA

## Background

Imaging patients with a pacemaker or AICD has always been taboo in the MRI environment. However, with current improvements in pacemaker lead and generator development along with very vigilant and knowledgeable personnel in pacemaker safety, MRI procedures can be implemented successfully. However, safe performance, not withstanding the risks, leads one to question if the results from the scans provide additional valuable clinical information to warrant the risk.

Hypothesis: We propose that MRI imaging patients with a pacemaker can be crucial to establish clinical diagnosis.

## Methods

A total of 25 patients were imaged on a GE CV/i Excite Version 12, 1.5 T system (GE, Milwaukee, WI). Three patients had an AICD, 4 patients had an AICD/Pacemaker, 2 patients had a single pacemaker lead and the remaining 16 patients had a complete pacemaker implantation. Each patient was performed in the dedicated Cardiac MRI Imaging Suite under the strict supervision of the Cardiologist. EP Lab personnel were present and reconfigured the pacemaker into an appropriate asynchronous mode under the guidance of the Cardiologist. The MRI scan sequences were selected such that the SAR level was lower or equal to 2.0 W/kg. to reduce additional heating to the device.

## Results

All patients completed the procedure with no adverse events and the pacemaker was interrogated after the procedure by EP Lab and reprogrammed under the direction of the Cardiologist. Impedance, thresholds, amplitudes and shock impedances were unchanged pre to post scanning. The average MRI scan time was 20±55min. Regarding the population, of the 25 patients imaged, 17 (68%) were neurology cases and 8(32%) were cardiac cases.

After reviewing the results from the 17 neurology cases and comparing the results from prior studies (CT, angio and/or myelogram) 14 (82%) out of the 17 patients benefited from having this procedure. 12 (70%) out of the 17 patients altered the diagnosis for a better outcome in patient care. The remaining 3(18%) patients did not show additional information that enhanced the diagnosis. The 8 cardiac cases were also compared to prior studies (heart cath, TEE, TTE and stress) and the outcome of all patients' diagnosis was shown to be enhanced by the MRI imaging. In 4(50%) of the 8 patients CMR altered the prevailing clinical diagnosis. Thus, a total of 22 patients (88%) benefited by enhancement or alteration of the original diagnosis while 3(12%) patients did not provide any additional information.

## Conclusions

The use of PM/AICD imaging in MRI remains controversial but as the lead/generator technology has improved, increased confidence in its use is found. Herein, we show that MRI procedures on carefully selected patients with pacemakers/AICD's are beneficial and substantially enhance or alter patient diagnosis. We propose that not only are Pacemakers/AICD's no longer taboo in the MRI environment but they can be markedly efficient with life-altering and life-saving consequences.

## Funding

None

